# Microbial Mg-rich Carbonates in an Extreme Alkaline Lake (Las Eras, Central Spain)

**DOI:** 10.3389/fmicb.2019.00148

**Published:** 2019-02-07

**Authors:** M. Esther Sanz-Montero, Óscar Cabestrero, Mónica Sánchez-Román

**Affiliations:** ^1^Department of Mineralogy and Petrology, Faculty of Geological Science, Complutense University of Madrid, Madrid, Spain; ^2^Department of Geology and Geochemistry, Faculty of Science, Vrije Universiteit Amsterdam, Amsterdam, Netherlands

**Keywords:** Mg-rich carbonates, Firmicutes, EPS, hydromagnesite, dolomite, microbialite, extreme lake Las Eras, decaying mats

## Abstract

This paper provides strong evidence for the contribution of the phylum Firmicutes in mediating the primary precipitation of Mg-rich carbonates (hydromagnesite, dolomite, magnesite, and nesquehonite) in recent microbialites from a highly alkaline and ephemeral inland lake (Las Eras, Central Spain). The carbonate mineral precipitation occurs sequentially as the microbial mats decay. Scanning electron microscopy (SEM) provided solid proof that hydromagnesite nucleation is initiated on the exopolymeric substances (EPS) and the microbial cells associated to the microbial mat degradation areas. The progressive mineralization of the EPS and bacterial cells by hydromagnesite plate-like crystals on their surface, results in the entombment of the bacteria and formation of radiating aggregates of hydromagnesite crystals. The hydrous phases, mostly hydromagnesite, were produced at a high percentage in the first stages of the microbial degradation of organic matter. When the availability of organic substrates declines, the heterotrophs tend to reduce their number and metabolic activity, remain dormant. At this stage, the anhydrous phases, dolomite and magnesite, nucleate on bacterial nanoglobules and/or collapsed cells. Evidence for the sequential formation of the Mg-rich carbonates trough the decay of organic matter by a fermentative EPS-forming bacterium isolated from the microbialites, *Desemzia incerta*, is drawn through a comparative analysis of carbonate formation in both natural and experimental settings. This study will help to constrain potential mechanisms of carbonate formation in natural systems, which are of fundamental importance not only for understanding modern environments but also as a window into the geologic past of Earth and potentially Mars.

## Introduction

A complex assemblage of hydrated metastable Mg-carbonate mineral phases, predominantly hydromagnesite [Mg_5_(CO_3_)_4_(OH)_2_⋅4H_2_O] are common in modern coastal and continental evaporitic and/or alkaline environments (Pueyo and Inglés, 1987; Last, 1992; Renaut, 1993; Wright, 1999; Coshell et al., 1998; Pérez-Rivarés et al., 2002; Desir et al., 2003; Power et al., 2007; Luzón et al., 2009; Last et al., 2010; Couradeau et al., 2013). Whilst the ancient sedimentary sequences are comprised of magnesite (MgCO_3_), which is considered the stable Mg-carbonate mineral (Zachmann and Johannes, 1989; Ordóñez and García del Cura, 1994; Salvany and Ortí, 1994; Sanz-Montero, 1996; Cañaveras et al., 1998, Melezhik et al., 2001; Sanz-Rubio et al., 2002; Sanz-Montero et al., 2006; Sanz-Montero and Rodríguez-Aranda, 2012). As with dolomite [CaMg(CO_3_)_2_], the abiotic precipitation of magnesite at low temperatures is kinetically inhibited (Vasconcelos et al., 1995; Vasconcelos and McKenzie, 1997; Sánchez-Román et al., 2009a, 2011a)as a consequence of the strong hydration of the Mg^2+^ ions (Slaughter and Hill, 1991). In contrast, the hydrated Mg-carbonate phases are often found associated with microbial mats in contemporary saline systems (Renaut, 1993; Braithwaite and Zedef, 1996; Coshell et al., 1998; Edwards et al., 2006; Power et al., 2007, 2009, 2017; Cabestrero and Sanz-Montero, 2018). Along this line, experimental investigations that examined mineral precipitation using microorganisms isolated from natural lakes (Thompson and Ferris, 1990; Power et al., 2007; Shirokova et al., 2013), have suggested the importance of cyanobacteria in promoting the formation of hydrated Mg-carbonates. The biologically induced precipitation of Mg-carbonates might occur at pH higher than 8.5 in aquatic environments with elevated magnesium concentrations (Thompson and Ferris, 1990). The precipitation of the Mg-rich carbonates being favored by the creation of micro-environments around the bacteria cells through the alteration of carbonate alkalinity and Mg^2+^ availability and acting as nucleation sites (Thompson and Ferris, 1990; Pontoizeau et al., 1997; Sánchez-Román et al., 2008, 2009b, 2011b; García Del Cura et al., 2014). Although microbes are known to mediate hydromagnesite formation under alkaline conditions, the processes and microorganisms involved in its precipitation are still poorly constrained. Even less well understood is the formation of a mineral assemblage comprised of Mg enriched carbonates, including dolomite and magnesite. To further complicate things, the physical, chemical and biological conditions vary considerably during different seasons in ephemeral lakes.

Lake Las Eras, in Central Spain, is an evaporitic highly alkaline inland lake where hydromagnesite and dolomite, among other carbonates, are precipitating (Sanz-Montero E. et al., 2013; Cabestrero and Sanz-Montero, 2018; Cabestrero et al., 2018). The lake is one of the few modern environments where hydromagnesite is a dominant precipitating mineral at the microbialite surfaces in association with dolomite. This work is aimed to study the mechanisms of formation of hydromagnesite and the associated Mg-rich carbonates in a complex and dynamic environment where microorganisms are subjected to different stress conditions (desiccation and exhausting supply). To further investigate the microorganisms involved in the precipitation of the mineral assemblage occurring in Las Eras microbialites, a carbonate forming bacterium was isolated from the carbonate layers within the microbial mat and identified by 16S rRNA gene sequencing as *Desemzia incerta* from the phylum Firmicutes. *D. incerta* culture experiments were performed at Earth’s surface sedimentary conditions. Mineralogical (XRD) and microscopical (SEM/EDS) analyses confirmed that the Mg-carbonate precipitates from the *D. incerta* laboratory culture experiments are similar to those associated to natural microbialites from both modern and ancient environments. In this work, we expanded the current knowledge on culturable diversity of carbonatogenic bacteria like Firmicutes by providing evidence for the precipitation of Mg-rich carbonates in alkaline lake environments that may have existed since the Archean, as well as in the geologic past of other planets such as Mars. Moreover, bacterial Mg-rich carbonate precipitation could be of importance in bioremediation of CO_2_ and Mg in extreme saline and alkaline environments.

## Geological Setting

Las Eras is an ephemeral lake, located in a closed-drainage area in the Duero Basin, Central Spain (41°12′1.04″N, 4°34′55.73″W). Las Eras with a maximum area of 0.1 km^2^ is a groundwater-fed, shallow basin (less than 0.6 m deep), affected by seasonal changes in temperature and in rainfall. Commonly from June to October, which corresponds with periods of low precipitation, the lake bed dries up and Las Eras converts to a playa (Sanz-Montero E. et al., 2013; Cabestrero and Sanz-Montero, 2018).

### Hydrochemistry

Las Eras is an alkaline (pH up to 11.3), brackish to saline (0.4–15.1 g⋅L^-1^) lake that have waters of the Na^+^-Cl^-^-(SO_4_^2-^)-(HCO_3_^-^) type (Sanz-Montero E. et al., 2013). The relative abundance of major cations in the water followed the trend of Mg^2+^ > Na^+^ > Ca^2+^, with a Mg^2+^/Ca^2+^ ratio up to 93 (Cabestrero and Sanz-Montero, 2018). Water analyses conducted by Cabestrero et al. (2018) showed that the pore water of the growing microbial mats was enriched in Mg^2+^, Cl^-^ and carbonate ions. Besides, the oxidation-reduction potential (ORP) in overlying water was negative, ranging from -177 to -61 mV.

### Carbonate Microbialites

The occurrence of actively mineralizing microbial mats in Las Eras lake bed was first documented by Sanz-Montero E. et al. (2013). Further studies showed that the composition of both the microbial communities and authigenic minerals seasonally changes as a response to fluctuations in the water lamina and in the geochemistry (Cabestrero and Sanz-Montero, 2018; Cabestrero et al., 2018).

The microbial mats harbor a complex and seasonally changing microbial community (Cabestrero et al., 2018) that consists mainly of photosynthetic cyanobacteria, predominantly *Oscillatoria-like* and *Phormidium-like*, the diatoms *Aneumastus sp*. and *Navicula sp*., and the charophyte *Chara canescens*, along with four major phyla (Firmicutes, Chloroflexi, Planctomycetes, and Actinobacteria) and four major classes of Proteobacteria (Alphaproteobacteria, Gammaproteobacteria, Deltaproteobacteria, and Betaproteobacteria).

**FIGURE 1:**
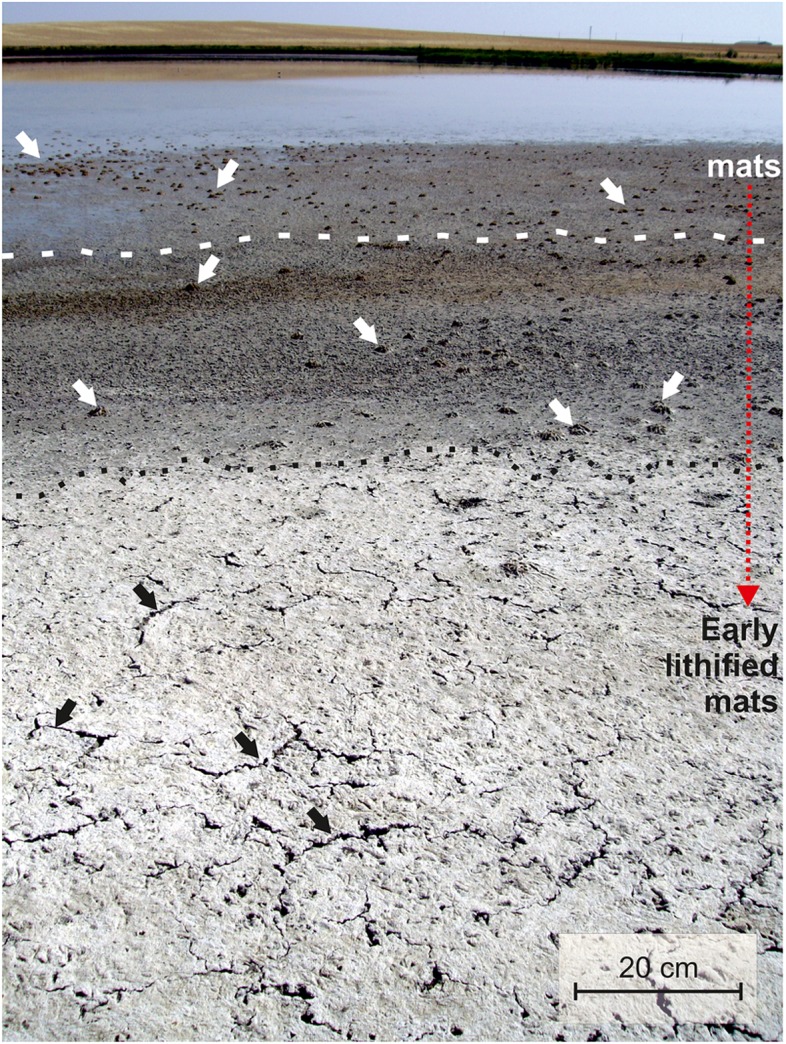
General view of Lake Las Eras surface which accommodates extensive microbial mats. Note mineralized microbialites (black dots) and incipient desiccation cracks (black arrows) in the desiccated littoral zone, and mat mounds (white arrows) in the wet zone. White dash line splits water saturated and unsaturated mats.

The resulting microbialites are composed of up to the 45% in mass of hydrous Mg-carbonates and 10% of dolomite (Sanz-Montero M. E. et al., 2013). These authigenic Mg-rich carbonates (hydromagnesite, nesquehonite MgCO_3_⋅3H_2_O and dolomite) are deposited mainly in the dry season as the water evaporates forming a white mineral crust that covers the decaying microbial mats on the ground (Sanz-Montero E. et al., 2013; Cabestrero and Sanz-Montero, 2018; Cabestrero et al., 2018; Figure 1). Apart from the detrital grains (clays, feldspars, quartz and carbonates) that account up to the 70%, and the aforementioned Mg-bearing carbonates, the microbialites are comprised of authigenic minerals that consist of fine-grained, calcite (CaCO_3_), natron (Na_2_CO_3_⋅10H_2_O), trona (Na_3_(CO_3_)(HCO_3_)2H_2_O), sulfates [thenardite Na_2_SO_4_, hexahydrite MgSO_4_ ⋅ 6H_2_O, bloedite Na_2_Mg(SO_4_)_2_⋅4H_2_O, gypsum CaSO_4_⋅2H_2_O, among others], sulfur and chlorides.

## Materials and Methods

Fieldwork in Las Eras basin (Figure 1) has been conducted for 17 days over the period 2012–2017 for sedimentological observations and sampling as well as to perform hydrochemistry determinations.

Water temperature, conductivity (salinity) and pH immediate measurements were taken in the field with a Hanna HI 9828 Sampler. Water samples were collected in duplicate directly from the lake. All samples were stored at 4°C. In order to examine the chemical composition of water, major ions were analyzed in filtered samples by ion chromatography at CAI of Geological Techniques, Geological Sciences Faculty, Complutense University of Madrid, Spain, using Dionex DX 500 Ion and METROHM 940 Professional IC Vario chromatographs. The carbonate (CO_3_^2-^) and bicarbonate (HCO_3_^-^) ion concentrations in the water were determined by titration (Rice et al., 2012).

### Mineralogy, Petrography and Morphology Analyses

Samples (0–10 cm depth) comprising biomass and mineral were collected following a transect from the edge toward the center of the lake when it was both dry and covered by a water lamina. The mineralogy of samples was determined by X-ray diffraction (XRD) analyses of powdered specimens on a Philips PW-1710 X-ray diffraction system operating at 40 kV and 30 mA, at 2°⋅min^-1^, with monochromated CuKα radiation. XRD spectra were obtained for 2θ to 66° 2q. X-ray diffraction analysis was performed by EVA Bruker software with automated search–match of the crystalline phases using the PDF2 database. This software was also used to semi-quantitatively determine the weight fraction of the mineral mixtures.

For preparation of thin sections, microbialite samples were dried, embedded and impregnated with Epofix resin. Optical examinations of thin sections were performed using an Olympus BX51 microscope.

For high-resolution textural analysis, thin sections and fresh broken surfaces of uncoated specimens were observed by an environmental scanning electron microscope (SEM-ESEM) FEI INSPECT in Non-destructive Techniques Service at Natural Sciences Museum of Madrid (MNCN), working at 30 kv at a distance of 10 mm, operating in high vacuum mode, and using secondary electron and backscatter detectors. In addition, gold-coated samples were analyzed with a Field Emission scanning electron microscope (FE-SEM) JSM 7600F in Centro Nacional de Microscopía Electrónica (UCM, Madrid, Spain) working also at 30 kv. Both microscopes were provided with Oxford Analytical-Inca X-ray energy dispersive system (EDS).

### Stable Isotope (C, O) Analyses

Eight representative samples of carbonates were analyzed for their isotopic composition (δ^13^C and δ^18^O). In five of these samples the carbon isotope composition of the organic matter associated to the carbonate mineral layers was also determined. The δ^18^O and δ^13^C values are given in per mil relative to Vienna Pee Dee Belemnite (VPDB). The crushed samples were evolved at 70°C using 100% phosphoric acid for 40 hours. All samples were prepared and analyzed, at least in duplicate. The analytical precision is generally ±0.10‰ for carbon and ±0.15‰ for oxygen. The δ^18^O compositions of the magnesium carbonates were corrected using the procedure described by Das Sharma et al. (2002).

### Bacterial Population Analyses

Evidence for microbially assisted formation of the hydromagnesite and associated carbonates is also drawn from laboratory analyses of samples containing microbial communities. The samples were collected into test tubes from the bed of the lake where thin mats were formed.

### Culture Medium

The culture medium (LE-1, modified from Sánchez-Román et al., 2007) used in our laboratory experiments has the following composition (%, w/v): 1% yeast extract; 0.5% proteose peptone; 0.1% glucose; and 2% NaCl. This medium was supplied with calcium and magnesium acetate to adjust the Mg/Ca molar ratio to 11. To obtain a 1 Bacto-Agar (Difco)-semi-solid medium, 20 g⋅L^-1^ was added. After the pH was adjusted to 7.0 with 0.1 M KOH, the medium was sterilized at 121°C for 20 min. Controls consisting of uninoculated culture medium and medium inoculated with autoclaved bacterial cells were included in all experiments.

### Microorganisms

The bacterial strain LE1 used for this study was isolated from the uppermost part of the microbial mat in Las Eras lake. This strain is a gram positive, alkaliphilic, fermentative microaerophilic bacterium (Stackebrandt et al., 1999). In order to obtain pure cultures of single strains, dilution series from the isolated samples taken from the microbial mat were inoculated onto Petri dishes containing the culture medium described above and incubated aerobically at 30°C. The Petri dishes were examined periodically to determine if the colonies were able to induce mineral precipitation.

The colonies forming a visible concentric corona of carbonate minerals were isolated. These pure strains were selected for phylogenetic 16S ribosomal deoxyribonucleic acid (rDNA) analysis (Centro de Astrobiología, INTA-CSIC, Madrid). The sequences obtained (ranging from 794 to 802 base pairs) were compared with the National Center for Biotechnology Information (NCBI) database. The results from the approximate phylogenetic affiliation revealed that the closest relative of strain LE1 is *Desemzia incerta* (Y17300) with 99.8% homology. This bacterium belongs to the Firmicutes order Lactobacillales (Stackebrandt et al., 1999), and has been identified in several environmental samples like compost (Ntougias et al., 2004), gold mine biofilms (Drewniak et al., 2008) and cold desert soil samples (Yadav et al., 2015).

### Geochemical Study

The activity of dissolved species and the degree of saturation in the natural and culture solutions assayed were determined using the geochemical computer program PHREEQC (Parkhurst and Appelo, 1999). The results from PHREEQC are presented in terms of the saturation index (SI) for each predicted mineral. SI is defined by SI = log (IAP/Ksp), where IAP is the ion activity product of the dissolved mineral constituents in a solubility product (Ksp) for the mineral. Thus, SI > 0 implies supersaturation with respect to the mineral, whereas SI < 0 means undersaturation.

Natural solution calculations were done considering the physicochemical values registered in the field together with the amount of major ions (Mg^2+^, Ca^2+^, Na^+^, K^+^, HCO_3_^-^, CO_3_^2-^, SO_4_^2-^, Cl^-^).

Culture medium calculations were performed applying the values of the added ions in the medium previously described above (g⋅L^-1^): Mg^2+^ = 3; Ca^2+^ = 0.45; Na^+^ = 11, Cl^-^ = 12, *P* = 0.15 (PO_4_^3-^ = 0.46) and NH_4_^+^ = 1.73. Total nitrogen in the culture media was determined by Kjeldhal’s method, while total phosphorus was determined colorimetrically in the nitrogen digests, generating the phosphomolibdic complex (Page et al., 1982). Neither bicarbonate nor other sources of inorganic carbon were added. Therefore the dissolve inorganic carbon (DIC) is controlled by air-solution exchanges. The amount of available C was adjusted equilibrating the solution to an atmospheric pCO_2_ value of 400 ppm.

## Results

### Characterization of the Microbialites

The precipitation of carbonate mineral phases takes place in the upper layers (up to 2 cm thick) of extensive lithifying microbial mats, during early spring and beginning of summer, when the lake desiccation triggers a high increase of cation and anion concentrations and a massive and rapid decay of biota (Figure 1).

#### Mineralogy

**FIGURE 2:**
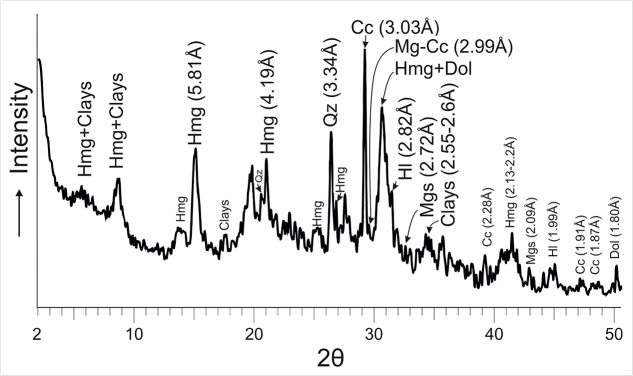
XRD showing the peaks of hydromagnesite-dypingite assemblage (Hmg), dolomite (Dol), magnesite (Mgs) and magnesian-calcite (Mg-Cc) as main authigenic Mg-carbonates of the microbialite. The other peaks correspond to Quartz (Qz), Calcite (Cc) and clays. Patterns used from the PDF2 database were: Hydromagnesite = 00-025-0513, Dypingite = 00-023-1218, Mica (clays) = 01-070-1869, Cc = 01-083-1762, Qz = 01-079-1910, Dol = 01-074-1687, Mgs = 01-086-2344, Mg-Cc = 01-086-2336, Hl = 01-075-0306.

The results of XRD analysis of representative samples of microbialites from 5 years period, 2012–2017, indicate the presence of a mixture of hydromagnesite and dypingite (referred to as Hmg hereafter) along with variable proportions of nesquehonite, dolomite, magnesite, eitelite [Na_2_Mg(CO_3_)_2_], trona, natron, calcite, and magnesian calcite (Figure 2). The Mg-rich carbonates represent up to 70% of the bulk mineralogy of the microbialites which are also comprised of a suite of sulfates, chlorides, phosphates and sulfur. Hydromagnesite, with a H_2_O/Mg ratio of 0.8, is the least hydrated of the hydrated metastable Mg-carbonates and the dominant mineral. On the opposite side, the most hydrated phase formed is the mineral nesquehonite (H_2_O/Mg ratio of 2). The H_2_O/Mg ratio in dypingite ranges 1–1.6.

#### Hydrogeochemical Modeling

Results of the geochemical modeling carried out using PHREEQC (Table 1) indicate that the lake water, with positive saturation indices, was always supersaturated with respect to the anhydrous carbonates dolomite (SI > 3.48), huntite (SI > 2.45), magnesite (SI > 1.18), calcite (SI > 0.41) and aragonite (SI > 0.26). These fluids were most of the time supersaturated in hydromagnesite and monohydrocalcite (avg. saturation of 3.32 and 0.35, respectively). On the contrary, the saturation indexes for nesquehonite (SI < -0.5) and the associated evaporites (not shown) were always negative.

**Table 1:** Saturation indices for selected minerals, as calculated by PHREEQC both for 17 water column (W. Col.) and pore water (Pore W.) samples collected during the 5 years period (2012–2017) and for culture medium.

#	Sample	Date	Sal	pH	Cc	Ara	Mhc	Dol	Hun	Nes	Hy	Mgs
1	W. Col.	Jan-12	11,0	9,6	1,01	0,86	0,18	4,78	5,66	-0,96	2,79	2,09
2	W. Col.	Nov-12	2,0	9,6	0,82	0,67	0,01	4,31	4,47	-1,33	0,69	1,77
3	W. Col.	Dec-12	2,4	9,9	0,84	0,69	0,03	4,72	5,64	-0,94	3,04	2,16
4	W. Col.	Mar-13	3,7	10,7	0,58	0,44	-0,22	3,48	2,45	-1,92	2,79	1,18
5	W. Col.	Mar-13	3,0	10,5	1,44	1,29	0,63	5,30	6,23	-0,93	4,10	2,15
6	W. Col.	May-13	3,2	10,8	1,64	1,50	0,81	5,61	6,92	-0,74	5,74	2,30
7	W. Col.	Jul-13	8,6	10,6	1,37	1,23	0,52	5,18	6,35	-0,84	5,22	2,18
8	W. Col.	Feb-14	4,3	10,4	1,49	1,34	0,70	5,33	6,03	-1,09	3,29	2,09
9	W. Col.	May-14	5,6	10,4	1,33	1,18	0,48	5,32	6,78	-0,67	5,34	2,35
10	Pore W.	Sep-14	5,1	9,1	0,86	0,71	0,00	4,94	6,64	-0,57	4,26	2,45
11	W. Col.	Sep-14	5,1	9,1	1,00	0,86	0,15	4,50	5,05	-1,14	1,02	1,88
12	W. Col.	Mar-15	5,8	11,1	0,79	0,64	-0,02	4,28	4,38	-1,37	3,81	1,76
13	W. Col.	May-15	7,1	9,9	1,37	1,23	0,54	5,25	6,38	-0,83	3,92	2,21
14	W. Col.	Mar-16	3,6	11,0	1,24	1,09	0,44	4,98	5,49	-1,19	3,97	1,99
15	W. Col.	Nov-16	5,5	9,1	1,81	1,67	0,99	5,15	5,11	-1,41	-0,09	1,65
16	W. Col.	Mar-17	6,7	9,7	0,41	0,26	-0,40	4,41	5,66	-0,78	3,43	2,30
17	W. Col.	May-17	12,7	9,5	2,00	1,85	1,16	5,89	7,07	-0,81	3,13	2,23
18	Culture	–	–	8,2	-0,27	-0,42	-1,13	1,76	-0,63	-2,63	-6,16	0,41


*Chemistry of the lake water varies depending on the season when the samples were collected. Sal, Salinity; Cc, Calcite; Ara, Aragonite; Mhc, Monohydrocalcite; Dol, Dolomite; Hun, Huntite; Nes, Nesquehonite; Hy, Hydromagnesite; Mgs, Magnesite.*

#### Isotopes

Isotopic composition (δ^13^C_V PDB_ and δ^18^O_V PDB_) of eight key microbialites samples with five different assemblages of carbonates (Table 2), indicate that the carbonates have depleted δ^13^C values ranging -3.56‰ to -1.22‰ (av. -2.71‰) and are enriched in ^18^O, with δ^18^O values of 1.34‰ to 6.37‰ (av. 4.56‰). The co-occurring organic matter has lower δ^13^C_org_ values (-23.15‰ to -21.02‰) (Table 2). The isotopic compositions of the bearing-nesquehonite samples tend to be enriched in ^13^C (δ^13^C av. -2.48‰) and in ^18^O (δ^18^O av. 5.08‰) relative to the assemblages without this mineral (δ^13^C av. -3.08‰, δ^18^O av. 3.68‰).

**Table 2:** Stable isotope values (C, O) of the carbonates and the organic carbon composing the microbialite.

Carbonate Assemblage	δ^13^C_carb_ ‰	δ^18^O_carb_ ‰	δ^13^C_org_ ‰	*XRD*				
				
				Dol %	Cc %	Hmg %	Nes %	Eit %
1	-1,22	5,42	-21,14	5	< 10	–	30	-
2	-1,33	6,16	–	< 5	5	20	< 10	-
2	-3,24	6,37	–	< 5	5	20	< 10	-
2	-3,32	2,21	-21,02	< 5	< 5	15	< 10	-
3	-3,30	5,24	-22,60	< 10	< 10	20	10	< 10
4	-3,20	1,34	-21,60	< 10	10	15	–	-
4	-2,48	5,66	-23,15	10	< 10	45	–	-
5	-3,56	4,05	–	< 10	< 10	–	–	-


*The semiquantitive analysis of the percentage values of each type of carbonates (X-Ray Diffraction – XRD) is also shown in the right side. Dol, Dolomite; Cc, Calcite; Hmg, Hydromagnesite/Dypingite; Nes, Nesquehonite; Eitelite, Eit. Carbonate assemblages are: 1, Dol+Cc+Nes; 2, Dol+Cc+Hmg+Nes; 3, Dol+Cc+Hmg+Nes+Eit; 4, Dol+Cc+Hmg; 5, Dol+Cc.*

#### The Microbialite Microfabrics From Las Eras Lake Sediment

**FIGURE 3:**
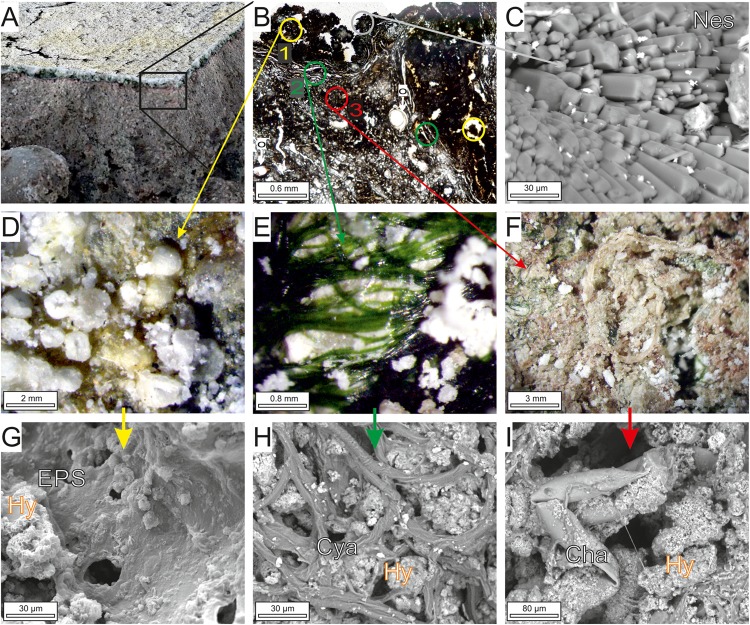
Sedimentary and microscopic features of the carbonate microbialites. **(A)** Cross-section through the microbialite showing the carbonate crust formed on two green and purple microbial layers that overlie buried microbial mats with abundant fenestral porosity due to gases and bioturbation. Hammer tip for scale is 1 cm. **(B)** Thin section microphotograph showing the internal structure of the microbialite with three distinct microfabrics that are arranged horizontally or in multilayered pockets: (1) thrombolitic (nodular) microfabric; (2) laminated microfabric with carbonate-coated filaments of green cyanobacteria; (3) purple-pigmented massive fabric that gradually downward grades to an older layer containing ostracods (o), charophyte remains and fenestral porosity. **(C)** Prismatic crystals of nesquehonite (Nes) on the mat surface. **(D)** Plan view of the hydromagnesite spherulitic structures formed on the yellow surface layer of the mat. **(E)** Laminar microfabric produced by hydromagnesite-coated filaments of green cyanobacteria. **(F)** Refractory charophyte remains embedded by purple-pigmented carbonates. **(G)** Nodules of hydromagnesite crystals with alveolar texture occur on both detrital grains and coccoid microbial cells encased in extracellular matrix (EPS), nearby the fenestral pores. **(H)** Hydromagnesite aggregates precipitated in the degraded cyanobacterial filaments and EPS. **(I)** Charophyte remains (Cha) partially mineralized by hydromagnesite. Note that **(D–F)** are microphotograps taken with binocular microscope; and **(C,G,H,I)** are SEM images.

The bed sediments comprise carbonate microbialites that overlie buried microbial mats with abundant fenestral porosity (Figure 3). The bulk of the carbonate production is associated to the three uppermost layers of the microbialite that are arranged horizontally or in multilayered pockets, each showing distinctive internal fabrics (Figure 3A,B). From the surface downward there are increasingly abundant carbonates. The most superficial layer contains hydromagnesitic spherulites coating a ground enriched in EPS colored by yellow pigments (Figure 3D). Underneath, a green layer with carbonate-coated bundles of filamentous cyanobacteria (Figure 3B,E) overlies a purple-pigmented and massive layer (Figure 3B,F). This layer downward grades to a gray bed containing ostracods, charophyte remains and fenestral porosity due to gases and bioturbation (Figure 3A,B). The uppermost layer consists of globular-topped columns of carbonate projected up to 1 mm upward from the surface (Figure 3B,D), exhibiting a thrombolitic, clotted microfabric (Figure 3B). Incipient to well-defined carbonate clots are formed at the top surfaces of the decaying mat when hydromagnesite aggregates precipitated on detrital grains and microorganisms encased in their extracellular matrix (EPS), preferentially nearby the fenestral pores (Figure 3G). Locally, prismatic nesquehonite crystals grow on the surface, associated to the hydromagnesite aggregates (Figure 3B,C). This upper layer is made up of a network of colorless filaments and green filamentous cyanobacteria, locally, ground populations of coccoids and diatoms are also abundant. The green layer is characterized by a laminated microfabric (Figure 3B,E) defined by hydromagnesite coated-cyanobacterial sheaths oriented parallel to the surface. The carbonate precipitation occurs within the EPS in between the emptied sheaths of filamentous cyanobacteria some of which have visible signs of degradation (Figure 3H). The laminated microfabric grades downward into a massive fabric where refractory, poorly defined charophyte rhizoids and oogonia are surrounded by purple carbonate precipitates (Figure 3F,I). The distinct purple pigmentation of this layer is due to the abundance of purple non-sulfur bacteria at this stage (Cabestrero et al., 2018).

**FIGURE 4:**
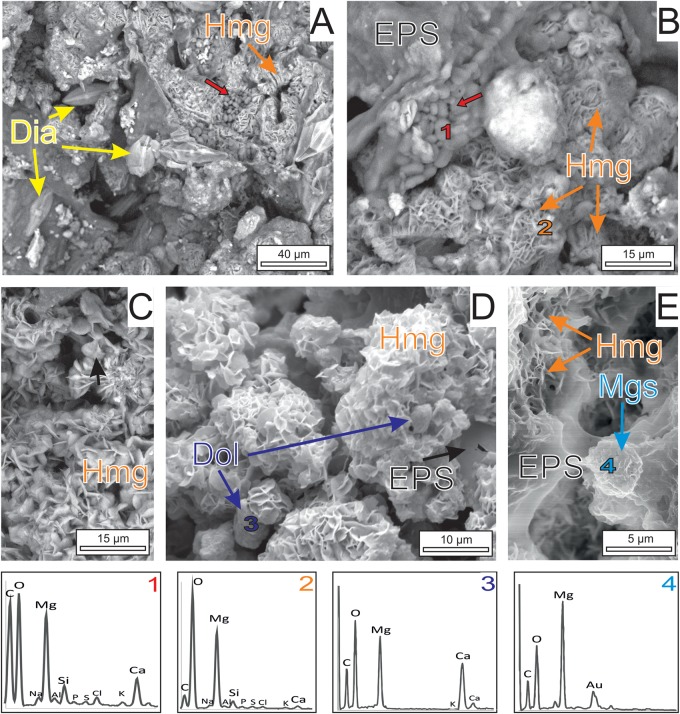
SEM images of the carbonate mineralization. **(A)** Graded transition between the mineralized and non-mineralized parts of the microbial mats. The hydromagnesite-dypingite association (Hmg) occurs as platelets radiating from the EPS, the diatoms (Dia) and the bacteria (red arrow). **(B)** Hmg crystals attached to mineralized microbial cells (spectrum 1) embedded in a EPS matrix. The cells completely mineralized by hydrous magnesium carbonates (Hmg) have globular forms displaying botryoidal morphologies (Spectrum 2). **(C)** Clusters of Hmg platelets showing dumbbell, pencil or star shapes. **(D)** Highly tight Hmg platelets deposited on EPS exhibiting a honeycomb arrangement. Note subidiomorph dolomite crystals (Dol, spectrum 3) embedded by the Hmg clusters. **(E)** Magnesite clusters (spectrum 4) resting on the EPS result from the aggregation of nanoparticles.

The precipitation of carbonates is coupled with the gradual degradation of the biomass of primary producers by heterotrophs (Figure 4). The initial hydromagnesite-dypingite deposits occur as plate-like crystals that radiate from the degraded diatoms, cyanobacterial sheaths and bacteria as well as the EPS embedding all of them (Figure 4A). The progressive deposition of carbonate crystals on the cells results in the entombment of the bacteria and the formation of dumbbell and spherulitic clusters of hydromagnesite (Figure 4B,C). The bacteria and the EPS contain high amounts of Mg, along with Ca, Si, and other minor elements (see EDS spectra in Figure 4). Except for Mg, the hydromagnesite coatings are depleted in all these elements. Similarly, the hydromagnesite deposited on the EPS surface form clusters of plate-like crystals that are slightly to highly tight, resulting in variable and concurrent morphologies such as rosette, dumbbell, random and fan shaped (Figure 4D). Well-defined hydromagnesite aggregates, up to 30 μm in diameter, typically have an alveolar texture (Figure 4). Associated to the hydrous magnesium carbonates, sparse dolomite and magnesite microcrystals rest on the EPS, surrounded by the hydromagnesite aggregates (Figure 4D,E). The dolomite and magnesite consist of composite crystals that result from the aggregation of crystalline nanoparticles (Figure 4E).

### Bacterially Mediated Carbonates From Culture Experiments

The potential of the isolated bacteria *D. incerta* to induce the carbonate precipitation was evaluated using liquid and solid media. Geochemical modeling (Table 1) indicate that the culture medium was undersaturated with the carbonates except for dolomite and magnesite which were slightly supersaturated (1.76 and 0.41, respectively). Hydromagnesite and nesquehonite should not precipitate from this fluid, as their saturation state were highly negative (-6.16 and -2.63).

**FIGURE 5:**
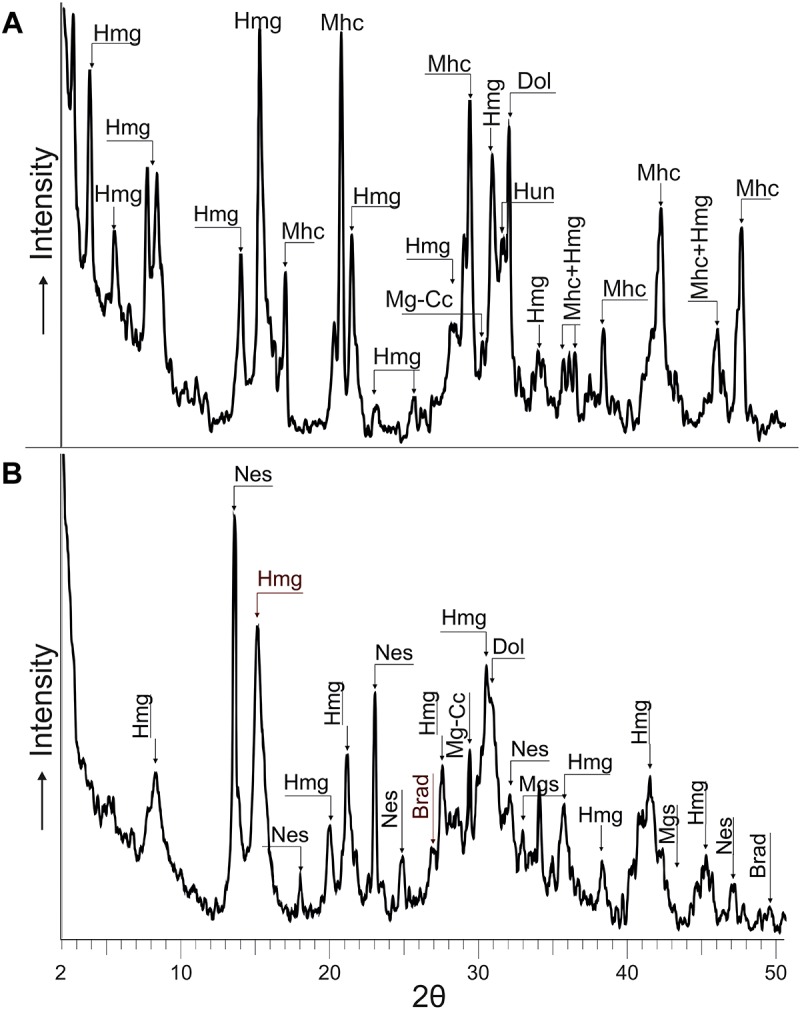
XRD of minerals recovered from liquid **(A)** and solid **(B)** culture samples. The precipitates were mainly hydromagnesite-dypingite assemblage (Hmg), monohydrocalcite (Mhc), dolomite (Dol), magnesium calcite (Mg-Cc), huntite (Hun), nesquehonite (Nes), magnesite (Mgs) and magnesium phosphate (Brad). See patterns used for mineral identification in Figure 2.

**FIGURE 6:**
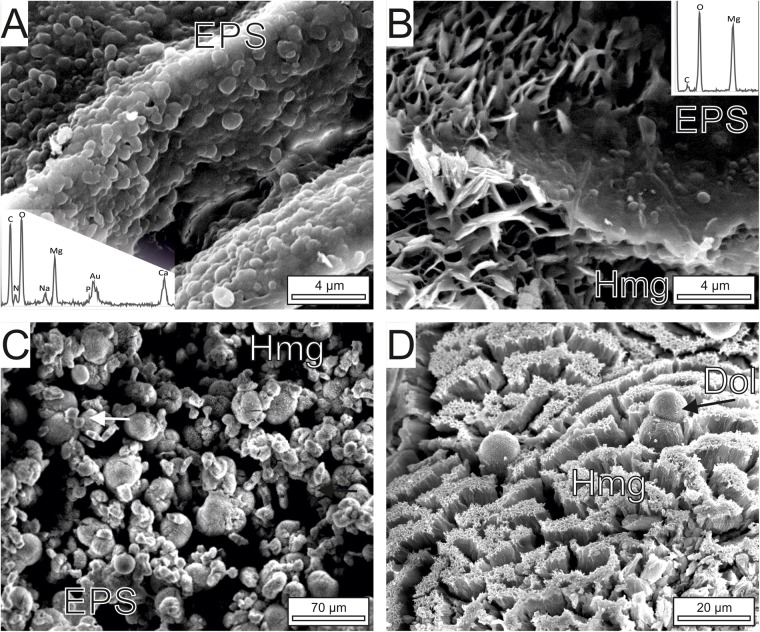
FE-SEM images of Mg-rich carbonates associated with the bacterial strain isolated from Las Eras. **(A)** Non-mineralized groups of the EPS forming bacterium. The EDS spectrum of the microbes shows that they are Mg-rich and contain Ca. **(B)** A smooth transition is observed between mineralized and non-mineralized EPS and microbial corpuscles. The mineral platelets are composed of hydrous Mg-carbonates (Hmg, see spectrum) that exhibit an alveolar arrangement. **(C)** General view of Hmg crystals associated to microbes and EPS remains that adopt spherulitic (globular), dumbbell and brush morphologies (arrowed). **(D)** Sparse dolomite spheroids (Dol) rest on the top of highly tight hydrous Mg-carbonate crystals.

**FIGURE 7:**
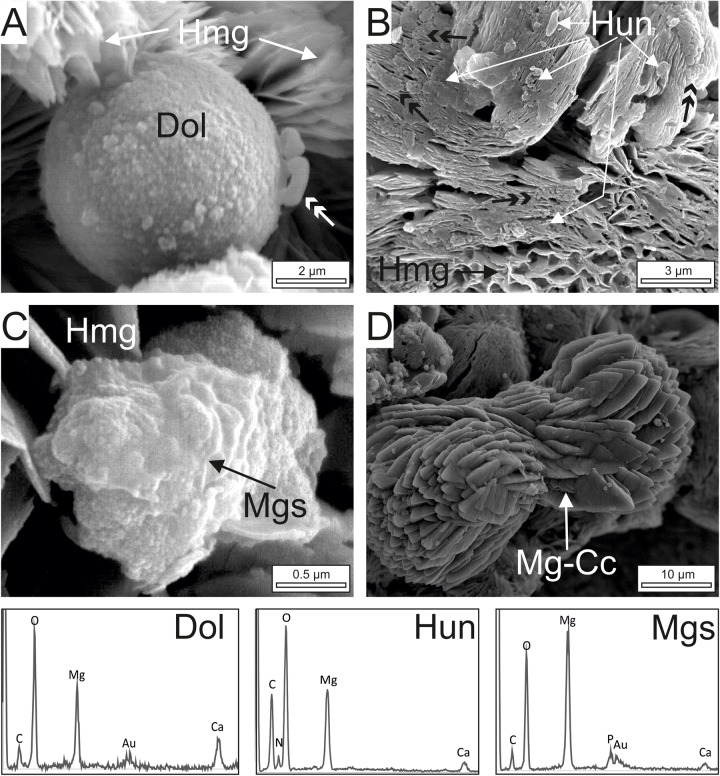
FE-SEM images and EDS-spectra of dolomite (Dol), huntite (Hun), magnesite (Mgs) and magnesian-calcite (Mg-CC) crystals associated with dypingite/hydromagnesite (Hmg). **(A)** Dolomite spheroid embedded by Hmg platelets. Note that dolomite consists of an aggregation of nanocrystals. Non-mineralized microbes on the surface are arrowed. **(B)** Clots of huntite plates lying on highly tight Hmg aggregates. The Hun crystals are similar in shape and size as well as in the type of clustering to the group of non-mineralized bacteria in the vicinity (double black arrows). **(C)** Composite magnesite crystal built up by nanoglobules showing poorly defined crystalline edges. **(D)** Well-formed euhedral Mg-calcite crystals exhibiting a dumbbell shape with sparse nanogranules on the surface.

After 15 days of incubation at 30°C, mineral precipitation was observed in both solid and liquid culture experiments. The recovered mineral precipitates were characterized by XRD (Table 3 and Figure 5) and by FESEM-EDS (Figure 6, 7). The mineralogical analysis of the precipitates indicates the presence of a variety of high Mg-carbonates in all the samples (Table 3). The monohydrated Ca-carbonate, monohydrocalcite (CaCO_3_⋅H_2_O), is also present in the experiments using liquid medium (Figure 5A), whilst minor amounts of Mg-calcite are recorded only in the solid culture containing the wider suite of carbonates (Figure 5B). Variable amounts of magnesian phosphates characterized as bradleyite are also present in both, solid and liquid media. The most abundant minerals are the hydrous Mg-carbonates formed by a dypingite-hydromagnesite assemblage (referred to as Hmg) that always occurs associated with dolomite and most rarely with Mg-calcite and magnesite. Nesquehonite is formed in the solid experiment (Table 3 and Figure 5B). In contrast, huntite was only detected in liquid medium (Figure 5A).

**Table 3:** Average XRD semiquantitative analysis of the minerals precipitated in *D. incerta* culture experiments (X-Ray Diffraction – XRD).

Culture	Hmg	Dol	Mgs	Nes	Hun	Mg-Cc	Mhc	Cc	MgP
Liquid	53	6	–	–	8	2	22	–	25
Solid	67	11	6	35	–	6.5	–	3	5


*Hmg, Hydromagnesite+Dypingite; Dol, Dolomite (poorly ordered); Mgs, Magnesite; Nes, Nesquehonite; Hun, Huntite; Mg-Cc, Magnesium Calcite; Mhc, Monohydrocalcite; Cc, Calcite; MgP, Magnesium Phosphates.*

FE-SEM images of the cultures (Figure 6, 7) show that the dominant carbonates (Hmg) occurs mostly as spherulitic aggregates formed by closely packed-crystalline platelets. The Hmg aggregates occur with different degrees of growth and packing and commonly show and alveolar arrangement. In the non-mineralized parts, the EPS-forming bacterium is still preserved and contain Mg and lesser quantities of Ca (Figure 6A). The transition between the mineralized and non-mineralized parts is gradual (Figure 6B,C). So, it is observed that the platelets nucleate radially on bacteria and the enclosing EPS (Figure 6B), creating poorly tight crystalline clusters with variable morphologies such as dumbbell, brushes and spherules (Figure 6C). The progressive aggregation of platelets into these initial groups causes the growth and coalescence of spherulitic clots (Figure 6C). Some bacteria and organic corpuscles are preserved on the outer surfaces of the Hmg platelets, where they occur associated to dolomite (Figure 6D, 7A), huntite (Figure 7B) magnesite (Figure 7C) and magnesian calcite (Figure 7D). Each mineral has been identified by their EDX spectra and their distinctive textures (Figure 7). Sparse, globular dolomicrite crystals are embedded in the Hmg ground (Figure 7A). Magnesite are composite crystals with poorly defined crystalline edges (Figure 7C). As the dolomite crystals, the magnesite is built up by nanoglobules. Huntite crystals consist of rounded-flaky crystals forming irregular sheets on the surface. The huntite flakes are similar in shape and size as well as in the type of clustering to the group of non-mineralized bacterial-like bodies that lie on the vicinity (Figure 7B). By contrast, dumbbell-shaped Mg-calcite is characterized by bigger and well-formed crystals with sparse nanogranules on the surface(Figure 7D).

## Discussion

The combination of a variety of mineralogic, microscopic and geochemical techniques with cultivation-independent molecular methods shows that degradation of microbial mats by the alkaliphilic heterotrophic bacterium *D. incerta* (phyllum Firmicutes) is actively involved in the formation of high Mg-carbonates including hydromagnesite, dypingite, dolomite, magnesite and nesquehonite in ephemeral alkaline lakes. Hydromagnesite, as the most stable phase of the hydrated magnesium carbonates, is the prevalent authigenic mineral. The precipitation occurs sequentially as the water evaporates and the microbial mats decay, the hydrated Mg minerals being the first to precipitate and the anhydrous phases (dolomite and magnesite), the latter. The sequence of precipitation of the high Mg-carbonates represents the progressive decline of organic matter until its exhaustion.

The inner structure of the resulting hydromagnesite microbialites is controlled by the layered structure of the microbial mat at the decaying stage, when carbonate minerals precipitate. With depth, the microfabrics vary from clotted (upper layer comprised of diatoms, coccoids, etc.) to laminated (green layer with some empty sheaths of cyanobacteria) and then, to massive (layer with pigments of the purple non-sulfur bacteria).

### Formation of Mg-rich Carbonates in Decaying Microbial Mats

Las Eras is one of the few modern environments where hydromagnesite is a dominant precipitating mineral in microbialites (e.g., Renaut, 1993; Braithwaite and Zedef, 1996; Russell et al., 1999; Power et al., 2009; Kazmierczak et al., 2011; Sanz-Montero M. E. et al., 2013). Moreover, the microbialites of Las Eras provides an exceptional study site in which hydromagnesite invariably occurs associated with dolomite, along with variable dypingite, nesquehonite and minor quantities of magnesite. The hydrochemistry of Las Eras is characterized by extremely high pH and Mg/Ca ratios (Cabestrero and Sanz-Montero, 2018). Such water chemistry creates high saturation conditions in the natural solutions for those Mg-rich carbonates, except for nesquehonite (Table 1). Even though the waters are supersaturated with respect to those carbonate mineral phases, spontaneous precipitation does not occur. Instead, the Mg-carbonate formation, including nesquehonite, only takes place within the microbial mats, mostly as the lake desiccates and the primary producers in the mats decay. Then, the precipitation processes occur rapidly.

Petrographic observations give evidence that the carbonate mineralization with predominant hydromagnesite and dolomite takes place on the decaying organic matrix and on the communities of microorganisms distributed in this matrix, including algae, cyanobacterial sheaths and microbial cells (Figure 3, 4). The distinct mineral spatial relationship within the mat explains the differences in mineralogy and textures. The hydrous Mg-carbonate precipitates as radiating plate-like crystals showing an alveolar microfabric, likely mimicking the honeycomb-alveolar EPS structure (Figure 4). This is in accordance with previous works showing that the decaying EPS is reorganized into alveoli that may act as mineral nucleation sites (DeFarge et al., 1996; Trichet et al., 2001). Our observations confirm that the surface of EPS in a hydrated stage tends to be smooth but acquires an alveolar structure as it is progressively transformed into carbonate from the degraded zones (Figure 6B). It is shown that the formation of radiating aggregates of hydromagnesite crystals is the results of the progressive deposition of plate-like crystals of hydromagnesite on the bacterial cells, which produce the entombment of the bacteria (Figure 4B). The precipitation of dolomite takes places as nanoglobules in microdomains of the hydromagnesite crystals where bacterial cells are also present. The dolomite crystals result of the progressive rearrangement of these corpuscles into larger, subidiomorph crystals. It is reiteratively seen that these nanoglobules referred to as nanobacteria-like particles are of great significance in carbonate formation (Folk, 1993), having been interpreted as particles produced by bacteria to avoid the cell entombment (Bontognali et al., 2008; Sánchez-Román et al., 2008).

The consistent negative δ^13^C_carb_ (from -1.33 up to -3.56‰) and high δ^18^O_carb_ (from 1.33 up to 6.37‰) values support that the precipitation of the hydromagnesite-rich assemblage is associated with the degradation of organic material as the lake water is subjected to evaporation at ambient temperature. Concurrently, δ^13^C_org_ values (av. -21.90‰) confirm the contribution of algae and microbes as sources of carbon (Meyers, 1994). The high δ^18^O values of the Eras microbialites are similar to the δ^18^O range reported for hydromagnesite-rich deposits associated to benthic microbial mats in modern alkaline lakes (Braithwaite and Zedef, 1996; Power et al., 2007), where the direct precipitation of hydromagnesite from surface water has not been observed either. As the evaporation alone is insufficient, the hydromagnesite nucleation was interpreted to be assisted by microbes (Braithwaite and Zedef, 1996; Power et al., 2007; Cabestrero and Sanz-Montero, 2018). By contrast, the biological formation of nesquehonite is more controversial. Its precipitation is attributed to evaporative processes on lake sediment surfaces where biofilms are absent (Power et al., 2007) or necessarily present (McLean et al., 1997; Sanz-Montero E. et al., 2013; Cabestrero and Sanz-Montero, 2018). In Las Eras, the nesquehonite-bearing carbonate assemblages are isotopically distinguishable by being slightly higher in ^13^C and in ^18^O (av. -2.48 and 5.08) relative to other carbonate paragenesis (av. -3.08 and 3.68, respectively), which confirms that the precipitation of nesquehonite is coincident with the evaporation of water highly enriched in ^18^O at the last stages of the evaporation processes. This agrees with recent hydrochemical simulation of Las Eras lake (Cabestrero and Sanz-Montero, 2018) showing that the saturation index of nesquehonite evolves to positive values when the solutions reach very high degrees of evaporation. The different ^18^O signal between nesquehonite and hmg mixtures may be explained in terms of their different structure, the hydromagnesite containing molecular water and hydroxyl (OH) groups, and the nesquehonite only molecular water (Glasser et al., 2016). Despite this, the negative δ^13^C_carb_ and δ^13^C_org_ values of nesquehonite-bearing paragenesis provide evidence for an organic source of carbon and a tool to determine the biogenicity of this mineral. The enrichment in ^13^C observed in the mineral associations containing nesquehonite with respect to other carbonate assemblages is coherent with the different occurrences of the main minerals in the microbial mats. The nesquehonite invariably occur at the microbial mat-water interface whilst the hydromagnesite is more abundant within the deeper layers in the zones subjected to degradation (Figure 3), where the relative contribution of carbon derived from organic matter is higher than in the surface.

### Firmicutes, a Novel Player in Mg-rich Carbonate Mineral Precipitation

It has been previously suggested that cyanobacteria photosynthesis play an important role in promoting the formation of hydrated-magnesium carbonates in highly alkaline aquatic environments (Power et al., 2007, 2009; Shirokova et al., 2013). However, our results do not support this mechanism of formation in Las Eras. Instead, SEM observations of natural samples show that the carbonate precipitation occurs on non-photosynthetically active decayed cyanobacteria, in association with heterotrophic bacteria (Figure 4). In addition, the experimental work carried out with single naturally occurring bacterium isolates has shown the capacity of a bacterium (identified as the bacterium Firmicutes *D. incerta*) to induce the precipitation of a suite of carbonates (dypingite/hydromagnesite, dolomite, nesquehonite, huntite, magnesite, Mg-calcite and monohydrocalcite) without the influence of cyanobacterial mechanisms/metabolisms. As occur in natural samples, the experimental results show that the mineralization extend in the biofilms of this EPS-forming bacterium from the zones of degradation. The precipitation of hydromangesite-dypingite, nesquehonite, monohydrocalcite, huntite and calcite occurs despite these minerals being undersaturated in the culture medium (Table 1). The preferential formation of these minerals over the supersaturated dolomite and magnesite show that bacteria can exert some control in the process of precipitation (Sánchez-Román et al., 2007, 2011a,b). The microorganisms and the EPS embedding the carbonate crystals contain Mg^2+^ and to a lesser extent Ca^2+^ and other cations (Figure 4, 6). The release of Mg and Ca through the bacteriolysis of the biomass may significantly increase the saturation levels inside the mats, favoring the precipitation of Mg-enriched phases (Cabestrero et al., 2018). The creation of micro-environments around the bacterial cells through the alteration of carbonate alkalinity and Mg^2+^ availability, and providing nucleation sites, leads to the precipitation of the Mg-rich carbonates (Thompson and Ferris, 1990; Pontoizeau et al., 1997; Sánchez-Román et al., 2008, 2011b; García Del Cura et al., 2014). In addition, the organic ligands on microbial cells may cause partial dehydration of the adsorbed Mg^2+^ that further facilitate the formation of Mg-bearing minerals by attracting the counter ions (Power et al., 2009, 2017).

The minerals grown in the laboratory bacterial cultures using pure isolates are quite similar in morphology and assemblage to those formed in natural microbialites, which confirms that Firmicutes are important players in the production of Mg-carbonates in the ephemeral alkaline Lake Las Eras. The capacity of Firmicutes to precipitate Ca-carbonates has been documented by Silva-Castro et al. (2015), but the involvement of this phylum in the formation of hydrous and anhydrous Mg-carbonates has not yet been shown. Firmicutes are one of the most abundant lineages in alkaline lakes, including Las Eras lake (Cabestrero et al., 2018 and references therein), which highlights the important role that some alkaliphic members of this phyla may have been playing in the formation of Mg-carbonates such as dolomite and magnesite since an early period in the Earth’s history.

### Sequence of Mineralization

Observation of carbonate minerals in natural and culture samples confirm that the hydrous and anhydrous Mg-carbonate mineral follow different mineralization pathways. First, the hydrous Mg-carbonates (dypingite/hydromagnesite) form massively in the decaying microbial mats. At the initial stages, poorly tight platelets of this mineral association precipitate on alveolar extracellular organic matrix and microbial cells. The precipitation of carbonates may result in the entombing of the bacteria and create dumbbell-shaped to rosette-like aggregates. The continued deposition of hydromagnesite produces continuous and densely packed aggregates at the expense of organic substrates that are drastically reduced by heterotrophs. When the availability of organic substrates declines, the heterotrophs tend to reduce their number and their metabolic activity (remain dormant) and likely produce clots of nanoglobules as evasion strategy. At this final stage, the anhydrous phases (dolomite, magnesite huntite, and other carbonates) precipitate either directly on the collapsed cells (as huntite) or in the nanoglobules they produce (dolomite and magnesite). Such mechanism of nucleation provides evidence for the primary and biologically aided formation of magnesite, excluding the rapid transformation of the hydrated phases into magnesite. As is the case of other type of bacteria and environments (Sánchez-Román et al., 2014, 2015), these finding corroborate that nanoglobules likely produced by Firmicutes act as nucleation sites for dolomite and magnesite. Few attempts to synthetize magnesite inorganically at low temperatures have succeeded (Deelman, 2012). By contrast, Power et al. (2017) documented the experimental precipitation of magnesite with the aid of microspheres, similar in size and in composition to bacteria, which supports that the microorganisms are instrumental for the formation of magnesite at low temperature.

Our results show that the individual isolates of Firmicutes are able to mediate the formation of different Mg-rich carbonates. The distinct mineral microbial relationship within the biofilm explains the differences in mineralogy and textures, being the anhydrous carbonates mineralizing the most resistant microbial corpuscles. The higher Ca contents in these corpuscles in relation to the EPS (Figure 4, 6) may explain these distinctive biomineralization styles. Thus, the chemistry of the cell walls may play an important role in the mineralization process as suggested by Al Disi et al. (2017).

### Crystalline Textures as Biomarkers: Implications for the Geological Record

The textural features and microfabrics of hydromagnesite formed in Las Eras microbial mats are similar to those reported in other modern alkaline lakes (Braithwaite and Zedef, 1996; Power et al., 2007), which provides a reliable criterion for assessing the biogenicity of these crystals. Russell and co-workers, in 1999, suggested the potential of preservation of the hydromagnesite microbialites in the rock record might be low. The latter observed that in Holocene sequences of Lake Salda Gölü (Turkey), the morphologies of the hydromagnesitic stromatolites were barely recognizable as they soon degenerate to porous, poorly lithified rocks. Those authors also assumed that the subsequent diagenetic dehydration of hydromagnesite to the more stable phase, magnesite, might have the effect of obliterating all signs of life in older rocks. However, the microfabrics and crystalline morphologies preserved in the Miocene magnesitic microbialites, described by Sanz-Montero and Rodríguez-Aranda (2012), give evidence that the destruction of microbial signatures does not necessarily occur. In contrast, the entire suite of crystal morphologies described in the modern counterparts is preserved in those lacustrine rocks, including dumbbell-shaped crystals, aggregates of radiating crystals grown from spheroidal nucleus, clusters with alveolar arrangements, etc., As in modern microbialites, the Miocene magnesitic microbialites are also composite crystals built up by the self-assembly of rods, platelets, fibers and microfossils that occasionally are associated to biofilms (Sanz-Montero and Rodríguez-Aranda, 2012). The good preservation of morphological signatures in this fossil analog confirm that the crystalline features of the hydrous magnesium phases are not obliterated by diagenetic transformations and, thus, can be used as biosignatures in the rock record.

## Conclusion

In highly alkaline lakes where cycles of wet and dry conditions occur, the formation of Mg-rich carbonates (hydromagnesite, dolomite, magnesite and hydrated phases) is controlled by the microbial decomposition of the biomass as the carbonate precipitation occurs sequentially until it is depleted. The hydrous metastable Mg-carbonates form first and the anhydrous minerals precipitate later, associated to the resistant and Ca-rich bacterial corpuscles. The hydrous and anhydrous Mg-carbonates have different crystalline textures, coherent with their distinct formation pathways. Thus, the availability of biomass is an important factor to be considered in biomineralization processes of Mg-rich carbonates in extreme environments. The biogeochemical interactions that control the precipitation of Mg-rich carbonates by bacteria could be of importance in bioremediation of CO_2_ and Mg in extreme alkaline environments.

The alkaliphilic heterotrophic Firmicutes are important players in the precipitation of Mg-carbonates in modern alkaline lakes, as they could have been in ancient environments. So, temporary lakes provide a modern analog to use in the interpretation of ancient alkaline and/or saline environments bearing Mg-carbonates. Clarification of how these Mg-rich carbonate phases are formed and stabilized may have broader implications on understanding the processes associated with microbial activity throughout geological time. Finally, this experimental study also provides potential biosignatures that may be useful to test Earth surface and extraterrestrial habitats for the presence and the biomineralization activity of bacteria similar to *D. Incerta.*

## Author Contributions

MES-M supervised the project, wrote the manuscript with support from the two co-authors, and carried out the fieldtrips as well as the microscopic observations with OC. OC manufactured the samples and characterized them with XRD, carried out the simulations by PHREEQC and drafted the figures. MS-R proposed and carried out the culture experiments aided by OC.

## Conflict of Interest Statement

The authors declare that the research was conducted in the absence of any commercial or financial relationships that could be construed as a potential conflict of interest.
